# Comparison of Efficacy and Safety of Anticoagulant Monotherapy and Combined Therapy of Anticoagulant and Antiplatelets in Patients With Stable Coronary Artery Disease and Atrial Fibrillation: A Meta-Analysis

**DOI:** 10.7759/cureus.29772

**Published:** 2022-09-30

**Authors:** Niharika Tentu, Aqsa Ijaz, Saima Batool, Rubba S Khan, Fathia Mohammed, Maryam H Khan, Qudsia I Sandhu, Neelum Ali

**Affiliations:** 1 Medicine, St. John’s Medical College, Bangalore, IND; 2 Internal Medicine, Allama Iqbal Medical College, Lahore, PAK; 3 Internal Medicine, Hameed Latif Hospital, Lahore, PAK; 4 Internal Medicine, Shifa International Hospital, Islamabad, PAK; 5 Internal Medicine, University of Gezira, Madani, SDN; 6 Internal Medicine, Rawalpindi Medical University, Lahore, PAK; 7 Medicine, Ghazi Khan Medical College Dera Ghazi Khan, Dera Ghazi Khan, PAK; 8 Internal Medicine, University of Health Sciences, Lahore, PAK

**Keywords:** meta-analysis, anti-platelet, oral anticoagulant, coronary artery disease, atrial fibrillation

## Abstract

It is still uncertain whether patients with atrial fibrillation (AF) and stable coronary artery disease (CAD) who require long-term oral anticoagulation (OAC) should also receive antiplatelet treatment (APT). This meta-analysis aims to compare the efficacy and safety of OAC alone with OAC plus APT in individuals with AF and stable CAD. The current meta-analysis was conducted as per the guidelines of Preferred Reporting Items for Systematic Reviews and Meta-Analyses (PRISMA) and the Meta-analysis of Observational Studies in Epidemiology (MOOSE). We performed electronic searches using PubMed, EMBASE, and Cochrane Library. The efficacy outcomes assessed in this meta-analysis included cardiovascular death, myocardial infarction, stroke (ischemic and hemorrhagic), and all-cause mortality. The safety outcome included major bleeding events. A total of five studies were included in the current meta-analysis enrolling 9199 patients with stable CAD and AF. Out of these five studies, three were observational and two were randomized controlled trials (RCTs). Our study showed no significant difference between two groups in the incidence of cardiovascular mortality (Hazard ratio {HR}: 0.86, 95% confidence interval {CI}: 0.59-1.25, I-square: 44%), myocardial infarction (HR: 1.21, 95% CI: 0.73-2.01, I-square: 0%), all-cause mortality (HR: 0.95, 95% CI: 0.76-1.19, I-square: 68%) and stroke (HR: 0.83, 95% CI: 0.61-1.12, I-square: 45%). However, lower incidence of major bleeding events in patients who received OAC alone as compared to patients who received a combination of OAC and anti-platelet (HR: 1.37, 95% CI: 1.18-1.580, I-square: 78%) were found. The current meta-analysis showed that OAC monotherapy is associated with a lower incidence of major bleeding events in patients with stable CAD and AF. It is also not associated with an increased risk of all-cause mortality, cardiovascular death, stroke, and myocardial infarction.

## Introduction and background

Atrial fibrillation (AF) is a disorder of heart rhythm that can cause irregular and rapid heartbeat [1]. Individuals with AF are more likely to have certain symptoms such as tiredness, shortness of breath, and palpitations [2]. AF can enhance the risk of heart failure by three times, the risk of death by 3.5 times, and the risk of stroke by five times [2]. One-third of people with AF also have coronary artery disease (CAD) [3]. Antiplatelet therapy (APT) and oral anticoagulant (OAC) are vital treatment options for AF patients with CAD who have gone through percutaneous coronary intervention (PCI) [4]. Antiplatelets function by preventing platelets from clumping together and building a clot, whereas anticoagulants prevent blood clotting by suppressing clotting proteins [5].

Individuals with CAD and AF usually receive a combination of OAC and APT. As per the guidelines of the European Society of Cardiology (ESC) and American College of Cardiology-American Heart Association (ACC-AHA), for AF patients with recent stent implantation or/and acute coronary syndrome (ACS), combined therapy of OAC and APT is ideal for 12 months but people with stable CAD and AF, the evidence is limited [6]. In persons with AF and stable CAD, adding antiplatelet treatment to oral anticoagulation was linked to a greater bleeding risk without a discernible improvement on ischemic end goals, according to several observational studies [7-9].

There has not been much research done on the optimal antithrombotic therapy for persons with AF and stable CAD. To reduce the risk of bleeding in this population, the 2016 European AF guideline and some professional consensus advise taking oral anticoagulation alone without any antiplatelet medication [10-11]. These guidelines are based on certain prospective and observational studies that have compared OAC alone with the combination of OAC and APT in patients with atrial fibrillation and stable CAD [7-9]. However, there are limited randomized control trials (RCTs) conducted on the topic [12-13]. In both the RCTs, Asian populations were involved and in these populations, the relative risk of bleeding and thrombosis is different compared to some other populations. Thus, it is important to perform a meta-analysis of available RCTs and observational studies. The aim of this meta-analysis is to compare the efficacy and safety of OAC alone with OAC plus single antiplatelets (SAPT) in individuals with AF and stable CAD.

## Review

Methods

The current meta-analysis was conducted as per the guidelines of Preferred Reporting Items for Systematic Reviews and Meta-Analyses (PRISMA) and the Meta-analysis of Observational Studies in Epidemiology (MOOSE).

Search Strategy

We performed electronic searches using PubMed, EMBASE, and Cochrane Library for searching relevant articles using the following keywords: “stable coronary artery disease”, “atrial fibrillation”, “oral anticoagulant”, “anti-platelets”, “anti-thrombotic” and “cardiovascular outcomes” without putting restrictions on year and language of publication. A reference list of all included studies was also searched to identify any relevant article.

Study Selection and Quality Assessment

Two authors reviewed the titles or abstracts of studies independently to identify the eligibility based on the inclusion and exclusion criteria. Randomized control trials (RCTs) and observational studies were eligible for inclusion if they compared OAC monotherapy with a combination of OAC and SAPT in patients with a medical history of AF and stable CAD. AF included permanent, long-standing persistent, persistent, and paroxysmal. Stable CAD is defined as coronary artery stenosis (≥ 50%) in one or more than one main coronary artery but did not require revascularization. In these patients, revascularization should be done at least six months before. We included studies that reported one of the three cardiovascular-related outcomes assessed in the current study (cardiovascular death, stroke, all-cause mortality, and myocardial infarction). We included studies that reported risk estimates for the investigated outcomes based on time‐to‐event data (hazard ratio {HR}). We excluded studies that assessed antithrombotic therapy in patients with AF and CAD after the percutaneous intervention.

Assessment of study quality was done by the two authors independently using Newcastle-Ottawa Quality Assessment Scale for observational studies and the Cochrane bias risk assessment tool for RCTs. An agreement between the two authors was mandatory for the final studies classification. The disagreement between the two authors was resolved via consensus

Outcome Measures

The efficacy outcomes assessed in this meta-analysis included cardiovascular death, myocardial infarction, stroke (ischemic and hemorrhagic), and all-cause mortality. The safety outcome included major bleeding events.

Data Extraction

Data from relevant articles were extracted using pre-designed data extraction sheets. Two reviewers independently extracted data from relevant articles. Following data were extracted from articles: a) first author b) year of publication c) study design d) groups e) sample size f) follow-up period g) participant characteristics f) outcomes. The disagreement between the two authors was resolved via consensus. One author entered the data into the Review Manager version 5.4.1 (The Cochrane Collaboration, London, UK).

Statistical Analysis

Review Manager version 5.4.1 was used to perform statistical analysis. Baseline characteristics of the pooled study population were given as mean and standard deviation for continuous variables and frequency and percentages for categorical variables. Because of the observational nature of most of the included studies in the current meta-analysis, the random effect model (inverse‐variance weighting) was used. Since the random-effect model considers the expected between-study heterogeneity when combining the results of separate studies, the random-effect model meta-analysis provides more cautious estimates than the fixed-effect model meta-analysis. A meta-analysis of hazard ratio (HR) was done and the results were presented with a 95% confidence interval (CI). Forest plots were generated for each of the outcomes assessed in this meta-analysis. Heterogeneity between studies was assessed using the Cochran Q test. To determine the degree of between‐study heterogeneity Higgins I-square statistics were calculated. P-values less than 0.05 were considered statistically significant.

Results

**Figure 1:**
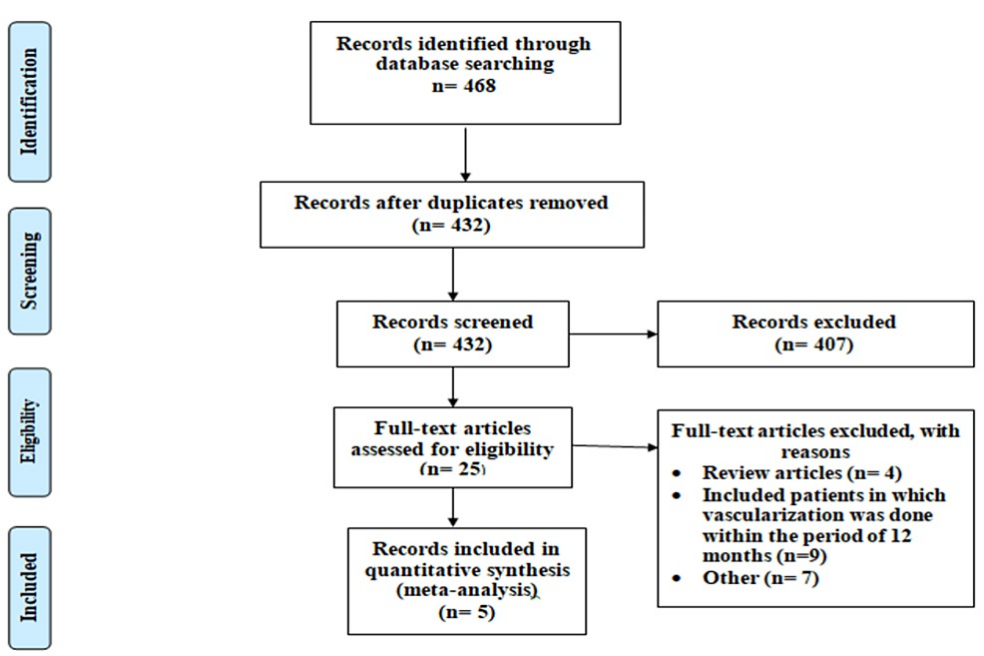
PRISMA flowchart of selection of studies PRISMA: Preferred Reporting Items for Systematic Reviews and Meta-Analyses

Figure 1 shows a PRISMA flowchart of the search and selection process used in the current meta-analysis. A total of 468 studies were identified through online database searching. After removing duplicates, 432 studies were eligible for the title and abstract screening. Out of 432 studies, 25 were revised for inclusion and exclusion criteria. In the end, five studies were included in the current meta-analysis enrolling 8249 patients with stable CAD and AF. Out of these five studies, three were observational and two were RCTs. One included study compared two kinds of OAC monotherapy (aspirin and clopidogrel) separately and they were used separately while performing pooled analysis.

The study characteristics of all included studies are shown in Table 1. Four-thousand-nine-hundred and fifty-nine were on monotherapy of OAC, while 3290 patients were on combination therapy of OAC and SAPT. The mean age of patients was 73.75 years, with a nearly 3:1 male-to-female ratio.

**Table 1:** Characteristics of included studies RCT: Randomized control trial; VKA: Vitamin K antagonist; DOAC: Direct oral anti-coagulant; MI: Myocardial infarction; SAPT: Single antiplatelet therapy; OAC: Oral anticoagulant * Study conducted by Lamberts et al. compared OAC monotherapy with OAC+aspirin and OAC+clopidogrel separately.

Author	Publication Year	Study Design	Groups	Sample Size	Type of OAC	Type of SAPT	Follow-up	Mean age (Years)	Males n(%)	Diabetes n(%)	History of stroke n(%)	History of MI n(%)
Fischer et al. [7]	2018	Retrospective Cohort	Monotherapy	127	VKA or DOAC	Aspirin or clopidogrel	2.8 Years	76	175 (68.90)	72 (28.35)	44 (17.32)	115 (45.3)
			Combined Therapy	127								
Lamberts et al. [8]*	2013	Retrospective Cohort	Monotherapy	950	VKA	Aspirin	1 Year	73.4	1600 (66.10)	358 (14.78)	449 (18.55)	1908 (78.8)
			Combined Therapy	1471								
			Combined Therapy	322	VKA	Clopidogrel	1 Year	73	226 (70)	60 (19)	67 (13)	141 (44)
													Lemesle et al. [9]	2017	Prospective cohort	Monotherapy	1481	VKA	Aspirin or clopidogrel	4 Years	73.2	1672 (71.20)	907 (38.9)	433 (18.7)	1266 (54.8)
Combined Therapy	866																								
Yasuda et al. [12]	2019	RCT	Monotherapy	1107	DOAC	Aspirin or P2Y12	2 Years	74.3	1750 (79.0)	927 (41.85)	323 (14.58)	777 (35.08)
			Combined Therapy	1108								
Nakano et al. [13]	2018	RCT	Monotherapy	344	VKA or DOAC	Aspirin or clopidogrel	2.5 Years	72.6	588 (85.20)	290 (42.03)	104 (15.07)	266 (38.55)
			Combined Therapy	346								

Overall, the quality of the included studies was high as shown in Table 2 for observational studies and Table 3 for RCTs. The overall quality of all included studies was high. 

**Table 2:** Risk of bias for observational studies

Study Id	Selection	Comparability	Outcome	Overall quality
Fischer et al. [7]	4	2	3	Good
Lamberts et al. [8]	4	2	3	Good
Lemesle et al. [9]	4	2	3	Good

**Table 3:** Risk of bias for RCTs

Study Id	Selection bias	Performance bias	Detection bias	Attrition bias	Reporting bias	Other biases	Overll quality
Yasuda et al [12]	Low	Low	Low	Low	Low	Low	High
Nakano et al [13]	Low	Low	Low	Low	Low	High	High

**Figure 2:**
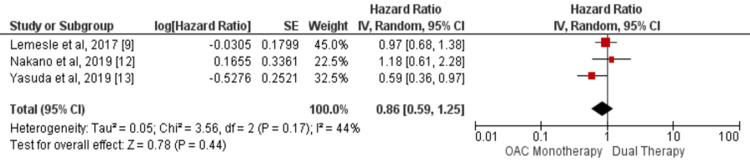
Pooled hazard ratio (HR) of cardiovascular mortality Pooled hazard ratio (HR) of cardiovascular mortality with a 95% confidence interval (CI) was calculated using a random effect model. The square shows the HR of each individual study while the diamond center shows the point estimate of pooled HR and the width denotes 95% CI of pooled HR. Sources: References [9,12-13]

**Figure 3:**
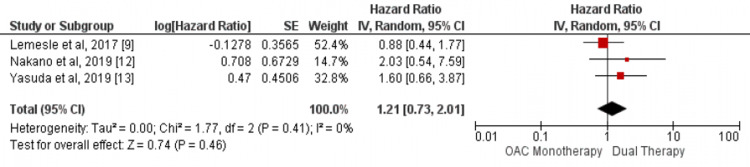
Pooled hazard ratio (HR) of myocardial infarction Pooled hazard ratio (HR) of myocardial infarction with 95% confidence interval (CI) calculated using random effect model. The square shows the HR of each individual study while the diamond center shows the point estimate of pooled HR and the width denotes 95% CI of pooled HR. Source: References [9,12-13]

**Figure 4:**
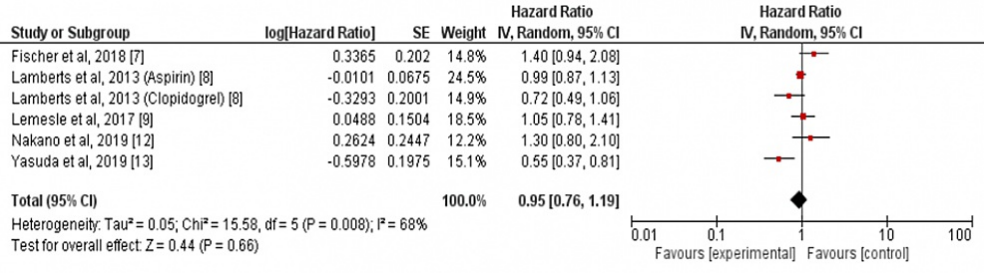
Pooled hazard ratio (HR) of all-cause mortality Pooled hazard ratio (HR) of all-cause mortality with 95% confidence interval (CI) calculated using random effect model. The square shows the HR of each individual study while the diamond center shows the point estimate of pooled HR and the width denotes 95% CI of pooled HR. Sources: References [7-9,12-13]

Our study showed no significant difference between the two groups in the incidence of cardiovascular mortality (HR: 0.86, 95% CI: 0.59-1.25, I-square: 44%) as shown in Figure 2 and myocardial infarction (HR: 1.21, 95% CI: 0.73-2.01, I-square: 0%) as shown in Figure 3. In addition, in terms of incidence of all-cause mortality, no significant differences were found in this meta-analysis between the two study groups (HR: 0.95, 95% CI: 0.76-1.19, I-square: 68%) as shown in Figure 4.

**Figure 5:**
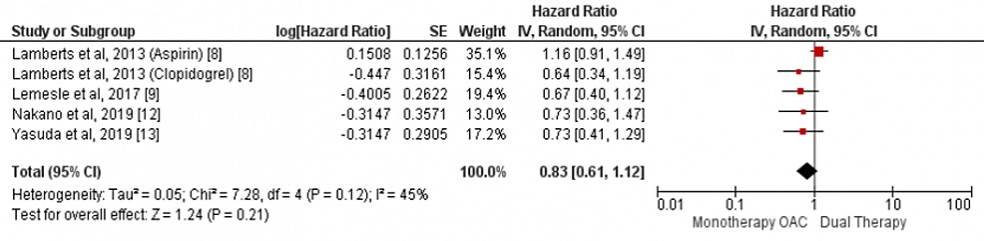
Pooled hazard ratio (HR) of stroke Pooled hazard ratio (HR) of stroke with 95% confidence interval (CI) calculated using random effect model. The square shows the HR of the individual study while the diamond center shows the point estimate of pooled HR and the width denotes 95% CI of pooled HR. Sources: References [8-9,12-13]

**Figure 6:**
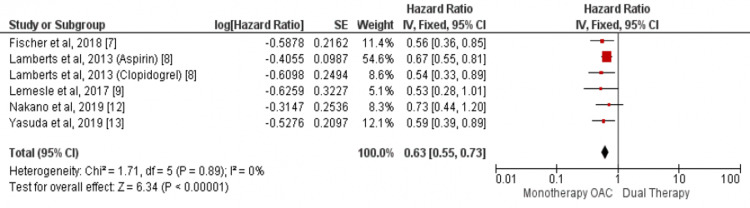
Pooled hazard ratio (HR) of major bleeding events Pooled hazard ratio (HR) of major bleeding events with 95% confidence interval (CI) calculated using random effect model. The square shows the HR of the individual study while the diamond center shows the point estimate of pooled HR and the width denotes 95% CI of pooled HR. Sources: References [7-9,12-13]

Our analysis showed no significant difference in regards to the incidence of stroke between the two study groups (HR: 0.83, 95% CI: 0.61-1.12, I-square: 45%) as shown in Figure 5. We found a lower incidence of major bleeding events in patients who received OAC alone as compared to patients who received a combination of OAC and anti-platelet (HR: 1.37, 95% CI: 1.18-1.580, I-square: 78%) as shown in Figure 6.

Subgroup Analysis

Table 4 shows the results of the subgroup analysis. In subgroup analysis based on the study design, all outcome variables closely mirrored the trends of the overall study results except for major bleeding events.

**Table 4:** Results of subgroup analysis RCT: Randomized control trial; HR: Hazard ratio; CI: Confidence interval * Significant at p-value<0.05

Outcomes	Subgroup	No. of studies	HR (95% CI)	I-square
All-cause mortality	RCT	2	0.84 (0.36-1.94)	62%
	Observational	4	1.01 (0.83-1.22)	25%
Cardiac mortality	RCT	2	0.81 (0.41-1.58)	-
	Observational	1	0.97 (0.68-1.38)	52%
Myocardial infarction	RCT	2	1.72 (0.83-3.59)	0%
	Observational	1	0.88 (0.44-1.77)	-
Stroke	RCT	2	0.73 (0.47-1.14)	0%
	Observational	3	0.85 (0.55-1.31)	65%
Major bleeding events	RCT	2	0.65 (0.46-0.93)*	0%
	Observational	4	1.60 (1.36-1.87)*	0%

Discussion

This meta-analysis aims to compare OAC monotherapy with a combination of OAC and SAPT in patients with atrial fibrillation and stable CAD. The findings of our meta-analysis showed that no significant difference was there in regards to cardiovascular mortality, stroke all-cause mortality, and myocardial infarction. However, pooled analysis of four observational studies and two RCTs showed that the incidence of major bleeding was significantly lower in patients who received OAC alone as compared to patients who received a combination of OAC and SAPT. These findings were unaffected by the study methodology.

Of the included studies in the current meta-analysis, there were RCTs conducted to compare the efficacy and safety of OAC monotherapy and combination therapy of OAC and SAPT in patients with stable CAD and AF. A study conducted by Matsumura-Nakano et al. found no significant difference in any of the outcomes assessed in the current meta-analysis [12]. This RCT has certain limitations. Firstly, the number of patients enrolled in the current meta-analysis was less than the intended population because of the early end of this study. The trial conducted by Yasuda et al. [13] found OAC more effective in reducing the incidence of all-cause mortality and cardiovascular mortality. The findings of this trial were not consistent with the findings of this meta-analysis. This trial was only conducted on the Japanese people and rivaroxaban was only used as OAC which raised a question about the external validity of this trial. The unexpected all-cause mortality and ischemia event reduction rates linked to OAC monotherapy in this study seem unexpected in the context of existing evidence. A large multi-national RCT is required to truly understand the impact of OAC monotherapy to understand the impact of possible confounding variables as well.

The EPIC-CAD trial is currently in progress, which is an investigator-initiated, multicenter, open-label randomized trial comparing the safety and efficacy of anticoagulant monotherapy with the combination of anticoagulant and antiplatelet drugs in patients with AF and stable CAD [14]. However, this trial is being conducted in South Korea only. Due to the lack of RCTs on the efficacy of monotherapy of CAD, this trial will contribute to the literature and a comparison with the trial conducted by Yasuda et al. will help professionals to understand the impact of monotherapy of OAC in a more detailed manner. In addition, it will also help in understanding the confounding variables affecting the response of treatments.

Even though guidelines usually recommended OAC monotherapy for patients with AF and stable CAD, the literature supporting the use of OAC monotherapy has been limited [15]. All of the included studies supported the point that OAC plus SAPT was not beneficial in the prevention of stroke and it also increased the risk of major bleeding events compared to OAC monotherapy [7-9,12-13].

Clinicians need to assess not just the risk of stroke, but also the bleeding and coronary events risk before making decisions for patients with AF and stable CAD. The best antithrombotic therapy should be chosen using a risk-factor-based methodology, according to the 2013 ACC guideline for AF [16]. The majority of AF patients should be assessed for stroke risk using the CHA2DS2-Vasc score, according to the 2011 ESC recommendation for AF [17].

The current meta-analysis has certain limitations. Firstly, the limitations of the studies we included, such as the observational character of several of the studies, greatly limit our findings. Secondly, our meta-analysis did not account for the use of variable cut-off for scores like HAS-BLED and CHADS2 square, used by the included studies. Thirdly, we were not able to analyze whether there is a certain group in which OAC plus SAPT treatment may be effective as we did not have access to patient-level data.

## Conclusions

The current meta-analysis showed that OAC monotherapy is associated with a lower incidence of major bleeding events in patients with stable CAD and AF. It is also not associated with an increased risk of all-cause mortality, cardiovascular death, stroke, and myocardial infarction. The beneficial impacts of OAC monotherapy were consistent across different types of study designs. In the end, large-scale randomized trials are required on high-risk populations comparing different OAC monotherapies to validate the findings of the current meta-analysis.
